# Effect of Resin, Calcium Silicate, and Silicone-Based Root Canal Sealers on Postoperative Pain: A Randomized Controlled Clinical Trial

**DOI:** 10.34172/joddd.42747

**Published:** 2026-03-30

**Authors:** Gizem Kadı, Esin Özlek

**Affiliations:** ^1^Department of Endodontology, Faculty of Dentistry, Altınbas University, İstanbul, Turkey; ^2^Department of Endodontology, Faculty of Dentistry, Van Yuzuncu Yil University, Van, Turkey

**Keywords:** Clinical trial, Postoperative pain, Root canal sealer

## Abstract

**Introduction:**

This study aimed to compare the effects of resin- (AH Plus), calcium silicate- (iRoot SP), and silicone-based (GuttaFlow Bioseal) root canal sealers on postoperative pain.

**Methods:**

This study included 84 patients with mandibular molars diagnosed with irreversible pulpitis. Root canal shaping procedures were completed using X-Smart Plus (Dentsply Maillefer, Ballaigues, Switzerland) endodontic motor and EndoArt (İnci Dental, İstanbul, Turkey) nickel-titanium rotary system up to #25 (30.06) shaping file for mesial root anals and #30 (0.6) shaping file for root distal canals. The patients were randomly divided into 3 groups based on the root canal sealer used (n=28): group 1: AH Plus, group 2: iRoot SP, and group 3: GuttaFlow Bioseal. Pain scores were recorded using a visual analog scale (VAS) before and after treatment at the 6th, 24th, 48th, and 72nd hours, and at the first week. Additionally, the frequency and timing of analgesic intake were recorded. The chi-squared test was used for categorical variables. The Kruskal-Wallis test was used to compare differences in non-normally distributed continuous data between groups. The Friedman test was used to compare pain scores over time within each group, and the Cochran’s Q test was used to analyze analgesic use.

**Results:**

No statistically significant differences in median VAS scores were found at various time intervals between the groups (*P*>0.05). Analgesic use distribution at the 6th (*P*=0.658) and the 24th (*P*=0.355) hours also did not show significant differences between the groups.

**Conclusion:**

Resin-, bioceramic-, or silicone-based sealers showed similar effects on postoperative pain.

## Introduction

 Postoperative pain, which starts within hours or days following endodontic treatment, is an undesirable condition for both the patient and clinician. The etiology of postoperative pain is multifactorial and is affected by factors related to the patient (age, gender, general health status, and psychological factors), tooth (tooth type, pulp and periradicular tissue status, presence of fistula, and presence of preoperative pain) and treatment procedures (length of operation, instrumentation, irrigation, obturation, number of sessions, and occlusal reduction)^1,2^ with reported incidence rates ranging from 3% to 58%.^3^ Among the predisposing factors are incomplete cleaning and shaping or obturation procedures.^4^ Root canal sealers can penetrate periapical tissues through apical foramina and lateral canals, or due to apical extrusion. Consequently, they may cause cytotoxic effects on periapical tissues, leading to inflammation, necrosis, and postoperative pain.^5^

 The epoxy-resin-based root canal sealer AH Plus (Dentsply, DeTrey, Konstanz, Germany) is widely used due to its low solubility, strong apical sealing, and satisfactory dentin adhesion.^6^ Recently, bioceramic-based root canal sealers have gained popularity as bioactive materials with less cytotoxicity compared to resin-based sealers. iRoot SP (BioCeramix, Vancouver, Canada), a bioceramic-based root canal sealer containing calcium silicate phosphate, is noted for its biocompatibility, superior physical properties, and the ability to maintain alkalinity for 14 days due to its calcium ion release properties.^7–9^

 GuttaFlow Bioseal (Coltene, Whaledent, Langenau, Switzerland) is a silicone-based root canal sealer with added calcium silicate particles. It is also a bioactive glass, with a finely ground gutta-percha powder and silicone-based matrix that disperses homogeneously upon mixing.^10,11^ It has a high water-absorption capacity, low solubility, and porosity. Additionally, it offers the benefits of alkalinization through calcium ion release and the formation of hydroxyapatite.^12,13^ Studies have reported that GuttaFlow Bioseal expands more than AH Plus after hardening, resulting in less leakage in root canal fillings and lower cytotoxicity.^10,14,15^

 Although several studies in the literature have focused on postoperative pain and its association with etiological factors and root canal sealers, to the best of our knowledge, no study has examined the effects of GuttaFlow Bioseal root canal sealer on postoperative pain. This study investigated and compared the effects of single-visit root canal treatments with AH Plus, iRoot SP, and GuttaFlow Bioseal sealers on postoperative pain 6, 24, 48, and 72 hours and one week after treatment. The null hypotheses were that there is no difference in the incidence and intensity of post-obturation pain among the AH plus, iRoot SP, and GuttaFlow Bioseal groups following single-visit root canal treatment.

## Methods

###  Study Design, Setting, and Sampling

 This randomized clinical trial was performed according to the Preferred Reporting Items for Randomized Trials in Endodontics (PRIRATE) 2020 guidelines. Ethical approval for the study was obtained from the Institutional Review Board and the Ethics Committee of Van Yuzuncu Yıl University (19/29.04.2021). The Consolidated Standards of Reporting Trials (CONSORT) guidelines were followed in this clinical trial, and the study protocol was registered on www.clinicaltrial.gov (Identifier: NCT05033093) and first posted on 26/08/2021. Voluntary patients were included in this study. All the patients signed an informed consent form after the objectives, procedures, benefits, and potential risks of the study were explained. The Power and Sample Size Calculation software version 3.1.2 was used to calculate the sample size.^16^ With 95% confidence, 80% test power, and f = 0.3476158 impact size, the total sample size was determined at n = 84. A total of 84 patients (28 per group) underwent single-visit root canal treatment performed by the same dentist.

###  Patient Selection and Pretreatment Assessment

 Healthy individuals aged 18 to 60, without systemic diseases, presenting with mandibular molar teeth exhibiting asymptomatic pulpitis perforating the vital pulp and requiring root canal treatment for preprosthetic purposes were eligible for inclusion. Additionally, teeth demonstrating positive responses to thermal and electric pulp tests were considered.

 Exclusion criteria included individuals who had used antibiotics, painkillers, or anti-inflammatory medications in the past seven days, pregnant or breastfeeding individuals, those with traumatic malocclusion or occlusal contact issues, teeth with visible periapical lesions on radiographs, material loss preventing the use of a rubber dam, teeth showing resorption, open apices, or calcification, teeth with gingival recession > 3 mm, deep pockets, mobility, or roots that were fractured or cracked.

 Patients underwent clinical examination, and periapical radiographs were obtained with the bisecting-angle technique. The gender (male/female), age, and preoperative pain scores of the patients were recorded. Preoperative pain assessment was conducted with the dentist to assess patient comprehension and cooperation with the pain scale. Patients unable to adequately engage with the scale were excluded from the study. Only one tooth per patient was included, and pulp vitality was assessed using an electric pulp testing device (Analytic Technology Corp., Redmond, WA, USA).

###  Randomization and Blinding

 Patient allocation to study groups was achieved using the envelope method, ensuring randomization regardless of age and gender, in a 1:1 ratio. The participating patients were blinded to their assigned groups as they received no information regarding group allocation. However, due to the study design, the clinician could not be blinded.

###  Clinical Interventions

 Local anesthesia was administered via inferior alveolar nerve block and buccal infiltration anesthesia using 2 mL of articaine hydrochloride containing 1:100,000 adrenaline (Ultraver DS fort; Haver, Istanbul, Turkey). Following rubber dam isolation, access cavities were prepared using a sterile round diamond bur. Tooth vitality was confirmed by observing bleeding during cavity preparation; teeth without bleeding were treated but excluded from the study. Working length (WL) was determined with an apex locator (Propex Pixi, Dentsply Maillefer) and confirmed to be 0.5‒1 mm shorter than the radiographic apex.

 The root canals were shaped using the crown-down technique with an X-Smart Plus (Dentsply Maillefer, Ballaigues, Switzerland) endodontic motor and EndoArt (Inci Dental, Istanbul, Turkey) nickel-titanium rotary system. In line with the manufacturer’s recommendation, all root canals were shaped with an entry file at 300 rpm and 3.5 Ncm torque, and with shaping files (#15 (0.4) and #20 (0.4)) at 300 rpm and 1.5 Nm torque, respectively. Then, the shaping procedure of mesial root canals was completed with finishing files #25 (0.6) at 300 rpm and 2.5 Ncm torque. In addition to mesial root canals, the distal root canals were shaped with finishing file #30 (0.6) at the same speed and torque settings. During shaping, when resistance was encountered, the files were removed from the root canal to prevent excessive torsion. The apical opening was checked with a #10K file, and root canal preparation was continued. These interventions were repeated until the targeted working length was achieved.

 During the shaping process, at each file change, root canal irrigation was performed with 2 mL of 5.25% NaOCl solution (Microvem AF, Istanbul, Turkey) for 1 minute. For the final irrigation of the root canals following the shaping process, 5.25% NaOCl, 17% EDTA (Imircryl, Konya, Turkey), 5.25% NaOCL, and saline solutions (5 mL of each) were used, respectively. For all irrigation applications, a 2-mL dental syringe and a #27G dental needle were used, both adjusted to be 2 mm shorter than the working length. After the final irrigation procedure, the root canals were dried with sterile paper points of matching diameter. Subsequently, the participants were categorized into three groups based on the type of root canal sealer used:

**Group 1:** AH Plus root canal sealer

**Group 2:** iRoot SP root canal sealer

**Group 3:** GuttaFlow Bioseal root canal sealer

 After obtaining radiographic control images with a master cone matching the last used file at working length for the single-cone technique, the selected gutta-percha cone (EndoArt, Inci Dental, Istanbul, Turkey) was coated with root canal sealer and inserted into the root canal. The root canal was then filled with the root canal sealer at the working length using back-and-forth movements. Following the application of glass-ionomer filling material (Ionoseal, VOCO, Cuxhaven, IOS) to cover the root canal opening as a standard procedure, permanent restoration was completed with a single-stage, self-adhesive, light-activated composite resin (Filtek Z250, 3M ESPE, St. Paul, Minnesota, USA). The root canal fillings were evaluated using radiography. All the patients were prescribed 400 mg of ibuprofen, with instructions to use it only in cases of severe pain.

###  Postoperative Pain Evaluation

 Postoperative pain was assessed using a visual analog scale (VAS).^9^ The patients were asked to record their perceived pain on the VAS at 6, 24, 48, and 72 hours, as well as one week after the procedure. Additionally, the patients were asked to record the number of analgesics used and the time of administration on the VAS scale. One week after the intervention, the patients were contacted by phone to obtain their recorded pain scores, which were then documented in the patient files. Patients who could not be reached by phone were excluded from the study.

###  Statistical Analysis

 The statistical analysis of the data was performed using SPSS 23. Normal distribution of the data was assessed using the Kolmogorov-Smirnov test. Categorical variables were compared between groups using the chi-squared test. The Kruskal-Wallis test was used to compare the data, which were not normally distributed, across the groups. The Mann-Whitney U test was used to compare the pain scores by gender. The Friedman test was used to compare intergroup pain scores over time. The Cochran’s Q test was used to compare analgesic use. The Spearman’s Rho Correlation Coefficient was used to assess the correlation between age and pain scores. Regarding the analysis results, quantitative data were presented as mean ± standard deviation and median (minimum-maximum), and categorical data were presented as frequencies (percentages). The results were evaluated at a significance level of *P* < 0.050.

## Results

**Figure 1:**
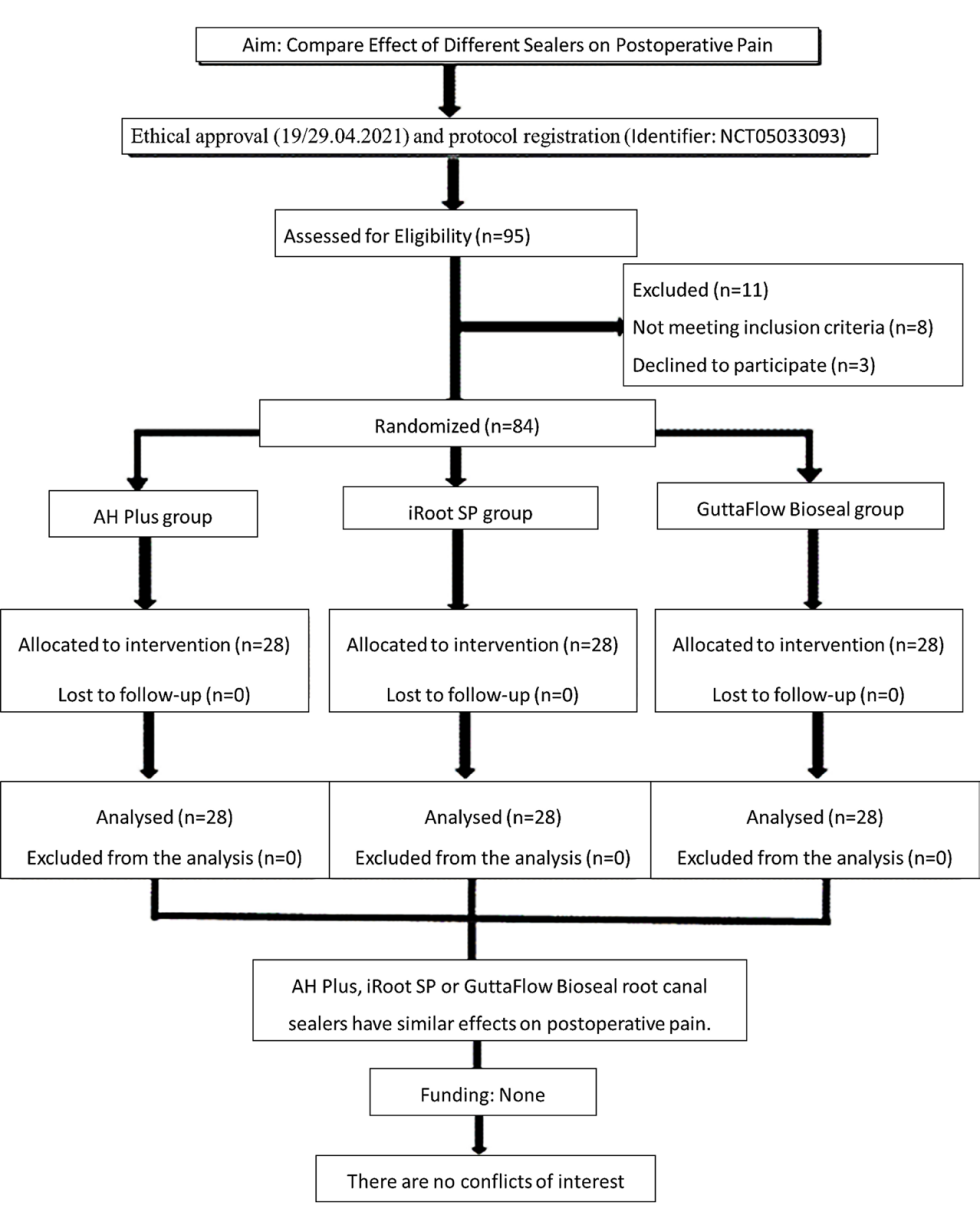
PRIRATE 2020 flowchart showing the flow of participants in the study

 Of the invited patients, 8 were excluded according to the established exclusion criteria, and 3 declined to participate. Consequently, they were excluded from the final analysis, leaving 84 participants (Figure 1). There were no statistically significant differences between the groups in gender (*P* = 0.953), age (*P* = 0.193), or preoperative pain scores (*P* = 0.766) (Table 1).

**Table 1:** Demographic and clinical features of the patients

	**AH Plus**	**iRoot SP**	**GuttaFlowBioseal**	**Total**	**Statistical Test**	**p**
Gender n (%)						
Female	15 (53.6)	16 (57.1)	16 (57.1)	47 (56)	*χ*^2^ = 0.097^b^	0.953
Male	13 (46.4)	12 (42.9)	12 (42.9)	37 (44)		
Age	27.57 ± 8.77	29.54 ± 7.33	31.68 ± 9.52	29.60 ± 8.65	*χ*^2^ = 3.295^a^	0.193
	24.50 (18.00‒51.00)	28.50 (18.00‒46.00)	31.50 (18.00‒57.00)	29.00 (18.00‒57.00)		
Preoperative pain	2.04 ± 2.55	2.07 ± 2.48	2.04 ± 1.93	2.05 ± 2.31	*χ*^2^ = 0.532^a^	0.766
	1.00 (0.00‒8.00)	0.50 (0.00‒7.00)	2.00 (0.00‒7.00)	1.00 (0.00‒8.00)		

^a^Kruskal-Wallis test statistic, ^b^chi-squared test statistic, mean ± standard deviation, median (min – max), frequency (percentage)

 The effects of AH Plus, iRoot SP, and GuttaFlow Bioseal root canal sealers on postoperative pain were not statistically significant (*P* > 0.05). Regarding the intragroup comparison of different times, the most severe postoperative pain was observed at 6 hours, and there was a statistically significant difference compared to other times (*P* = 0.001) (Table 2).

**Table 2:** Comparison of pain VAS scores between and within groups

	**AH Plus**	**iRoot SP**	**GuttaFlowBioseal**	**Total**	**Statistical Test**^b^	**p**
Preoperative pain score	2.04 ± 2.55	2.07 ± 2.48	2.04 ± 1.93	2.05 ± 2.31	*χ*^2^ = 0.532	0.766
	1.00 (0.00‒8.00)^bc^	0.50 (0.00‒7.00^)cd^	2.00 (0.00‒7.00)^b^	1.00 (0.00‒8.00)		
6 hrs pain score	2.86 ± 2.92	2.68 ± 2.39	2.04 ± 1.60	2.52 ± 2.36	*χ*^2^ = 0.736	0.692
	2.50 (0.00‒10.00)^b^	2.00 (0.00‒10.00)^c^	2.00 (0.00‒6.00)^b^	2.00 (0.00‒10.00)		
24 hrs pain score	1.43 ± 2.15	1.43 ± 1.87	0.89 ± 1.40	1.25 ± 1.83	*χ*^2^ = 1.317	0.518
	0.00 (0.00‒8.00)^ab^	1.00 (0.00‒6.00)^bc^	0.00 (0.00‒5.00)^ab^	0.00 (0.00‒8.00)		
48 hrs pain score	0.46 ± 1.29	0.61 ± 0.92	0.50 ± 1.17	0.52 ± 1.12	*χ*^2^ = 4.619	0.099
	0.00 (0.00‒5.00)^ac^	0.00 (0.00‒4.00)^abd^	0.00 (0.00‒4.00)^a^	0.00 (0.00‒5.00)		
72 hrs pain score	0.21 ± 0.83	0.07 ± 0.38	0.11 ± 0.42	0.13 ± 0.58	*χ*^2^ = 0.423	0.809
	0.00 (0.00‒4.00^)a^	0.00 (0.00‒2.00)^ab^	0.00 (0.00‒2.00)^a^	0.00 (0.00‒4.00)		
1 week pain score	0.00 ± 0.00	0.00 ± 0.00	0.00 ± 0.00	0.00 ± 0.00	---	---
	0.00 (0.00‒0.00)^a^	0.00 (0.00‒0.00)^a^	0.00 (0.00‒0.00)^a^	0.00 (0.00‒0.00)		
Statistical Test^a^	*χ*^2^ = 68.959	*χ*^2^ = 68.151	*χ*^2^ = 72.533			
p^a^	< 0.001	< 0.001	< 0.001			

^a^ *χ*^2^: Friedman test statistic, _b_ *χ*: Kruskal Wallis test statistic, a-d: No difference between times with the same letter within groups, mean ± standard deviation, median (min – max)

 There was no statistically significant difference in analgesic use between the AH Plus, iRoot SP, and GuttaFlow Bioseal groups at different time intervals (6th, 24th, 48th, 72nd hours, and one week) (*P* > 0.05) (Table 3). Although there was no statistically significant difference in analgesic use across time points in the iRoot SP group, the highest incidence of analgesic use was observed at the 6th hour in the AH Plus and GuttaFlow Bioseal groups, with a statistically significant difference (*P* < 0.05).

**Table 3:** Comparison of the use of analgesics between and within groups

	**AH Plus**	**iRoot SP**	**GuttaFlowBioseal**	**Total**	**Statistical Test**	**P**_b_
6 hrs analgesic intake						
No	23 (82.1)^a^	25 (89.3)	25 (89.3)	73 (86.9)	*χ*^2^ = 0.837	0.658
Yes	5 (17.9)	3 (10.7)	3 (10.7)	11 (13.1)		
24 hrs analgesic intake						
No	26 (92.9)^ab^	27 (96.4)	28 (100)	81 (96.4)	*χ*^2^ = 2.074	0.355
Yes	2 (7.1)	1 (3.6)	0 (0)	3 (3.6)		
48 hrs analgesic intake						
No	28 (100)^b^	28 (100)	28 (100)	84 (100)	---	---
72 hrs analgesic intake						
No	28 (100)^b^	28 (100)	28 (100)	84 (100)	---	---
1 week analgesic intake						
No	28 (100)^b^	28 (100)	28 (100)	84 (100)	---	---
Test statistic	Q = 16.0	Q = 8.5	Q = 12.0			
P_a_	0.003	0.075	0.017			

*χ*: chi-squared test statistic, Q = Cochran’s Q test statistic, a-b: There is no difference between tenses with the same letter. Bold numbers are P values for Cochran’s Q test

## Discussion

 Postoperative pain following root canal treatment is commonly attributed to acute inflammation in periapical tissues.^17^ The success of the root canal treatment depends not only on the efficacy of the clinical procedures or biological outcomes but also on the optimization of the patient comfort by minimizing postoperative pain.^18^ Consequently, this study aimed to assess the impact of three different root canal sealers (AH Plus, iRoot SP, and GuttaFlow Bioseal) used in the endodontic treatment on postoperative pain. According to the findings, the effects of AH Plus, iRoot SP, and GuttaFlow Bioseal root canal sealers on postoperative pain were not statistically significant, and the null hypothesis was accepted.

 Epoxy-resin-based root canal sealers are associated with significant cytotoxic effects on periradicular tissues by inducing inflammatory mediators, including cyclooxygenase-2, nitric oxide synthase, receptor activator of nuclear factor kappa B ligand, and reactive oxygen species.^19,20^ Additionally, AH Plus has been linked to the release of monomers, such as bisphenol A and diglycidyl ether, which contribute to its cytotoxicity.^21^ Moreover, it has been reported to increase the expression of pro-inflammatory cytokines such as interleukin-6 and interleukin-8, and to significantly decrease mitochondrial activity.^22^ The literature contains only a limited number of studies on the effects of bioceramic-based root canal sealers on postoperative pain. To our knowledge, no other study has investigated the effect of silicon-based root canal sealers on postoperative pain, preventing direct comparison of our findings. Studies have shown that AH Plus exhibits higher cytotoxicity than both bioceramic-based^8^ and silicone-based^14,23^ root canal sealers, and causes more neurotoxic effects.^24^ Consistent with our findings, Ateş et al.^25^ reported that AH Plus caused more postoperative pain than iRoot SP; Aslan and Donmez^26^ found that AH Plus resulted in more postoperative pain than EndoSequence BC Sealer; Tan et al.^27^ reported that AH Plus led to more postoperative pain than Total Fill; Shim et al.^28^ indicated that AH Plus caused more postoperative pain than Endoseal MTA; and Ferreira et al.^29^ reported that AH Plus caused more postoperative pain than MTA Fillapex and Endofill.

 The physical and chemical properties of root canal sealers, such as pH and consistency, are considered crucial factors influencing the severity of postoperative endodontic pain.^30,31^ The alkaline environment caused by root canal sealers plays a vital role in periapical recovery by inhibiting osteoclastic activity and promoting hard tissue formation through alkaline phosphatase activation.^32^ It was reported that in 7 days, the pH increased up to 8.17^32^, 8.75^30^, and 9.21^12^ with AH Plus, iRoot SP, and GuttaFlow Bioseal, respectively. It was reported that GuttaFlow Bioseal released 3.80 mg/L Ca^2+^ions at the end of the 14th day, which increased to 3.89 mg/L at the end of the 28th day.^9^ The Ca^2+^ion release increased to 2.96 mg/L within 14 days, but this level was not sustained at the end of the 28th day and dropped to 2.015 mg/L.^30^ In contrast, AH Plus root canal sealer exhibited significantly less Ca^2+^ion release ( < 1.00) compared to other sealers.^31,33^ Although there was no statistically significant difference in postoperative pain between the root canal sealers preferred in this study, AH Plus root canal sealer induced more postoperative pain. We believe this was related to differences in pH resulting from the amount of Ca^2+^ions released, with lower pH inducing greater postoperative pain.

 iRoot SP and GuttaFlow Bioseal are resin-free, biocompatible root canal sealers that contain bioactive components and have shorter setting times than AH Plus. The reported mean setting times were 4 hours for iRoot SP,^34^ 17 minutes for GuttaFlow Bioseal, and 8 hours for AH Plus.^33^ Prolonged setting times of root canal sealers may impact biocompatibility and the potential release of cytotoxic byproducts during the hardening reaction.^35^ Therefore, bioactive root canal sealers are expected to induce much less postoperative pain than epoxy-resin-based AH Plus.^27^ This explains why postoperative pain incidence was numerically highest in the AH Plus group and lowest in the GuttaFlow Bioseal group at the 6th hour, despite no statistically significant difference observed in this study.

 Studies have demonstrated the importance of non-steroidal anti-inflammatory agents in relieving postoperative pain following endodontic treatment.^36^ Consistent with Aslan et al.’s^27^ study, we prescribed 400-mg ibuprofen (Brufen) to all patients upon treatment completion. Additionally, we recommended analgesic use only in cases of severe pain to prevent regular drug use from biasing postoperative pain assessments. Regardless of the group, the evaluation of all patients showed that 13.1% used analgesics at the 6th hour, 3.6% at the 24th hour, and none at other times. According to the groups, there was no statistically significant difference between the distribution of the analgesic use frequency at the 6th and 24th hours. However, the quantitative evaluation showed that 11 patients received medication at the 6th hour, with 5 in the AH Plus group, 3 in the iRoot SP group, and 3 in the GuttaFlow Bioseal group. Additionally, three patients used analgesics at the 24th hour, with two in the AH Plus group and one in the iRoot SP group. Additionally, three patients used analgesics at the 24th hour, with two in the AH Plus group and one in the iRoot SP group. This underscores the highest incidence of analgesic use at the 6th hour, consistent with findings from other postoperative pain studies, potentially attributed to local anesthetic injection, pressure from rubber dam clamps, or discomfort from prolonged mouth opening.^26,27^

 In this study, the mean postoperative pain scores at the 6th and 24th hours were 2.86 and 1.43, respectively, in the AH Plus group; 2.68 and 1.43, respectively, in the iRoot SP group; and 2.04 and 0.89, respectively, in the GuttaFlow Bioseal group. There was no statistically significant difference in the mean VAS scores between the study groups at different times (*P* > 0.05). We believe that these differences in the incidence of postoperative pain and drug use to the varying cytotoxic potentials of the sealers.

 When assessing time-dependent postoperative pain scores, the highest score was observed at the 6th hour in all groups and gradually decreased thereafter. Regardless of the group, all patients’ postoperative pain mean scores were 2.52, 1.25, 0.52, 0.13, and 0 at the 6th, 24th, 48th, and 72nd hours and at the end of the first week, respectively.

 Consistent with previous studies, the most severe postoperative pain was observed at the 6th hour and halved by the end of the first day.^27,37,38^ Short-term postoperative pain is believed to be related to the induction of reactive oxygen species (ROS) formation caused by the leakage of non-polymerized components of root canal sealers during the first 24 hours of setting.^39^ Over time, the incidence of postoperative pain significantly decreased, with none of the patients reporting pain at the end of the first follow-up week, and none requiring retreatment. These findings align with several postoperative pain studies.^29,40-42^

 Regarding gender’s effects on postoperative pain, the literature presents conflicting results. Some studies report longer duration and higher incidence of postoperative pain among females,^2,43^ while others suggest longer duration among males compared to females.^44^ Similarly, studies examining the correlation between age and postoperative pain offer inconsistent findings, with some reporting increased pain with age,^45,46^ and others suggesting a decrease.^47,48^ Despite the lack of consensus in the literature,^27,29,49,50^ our study found no significant correlation between postoperative pain and gender or age.

 The procedural stages of root canal treatment make it impossible for the endodontist to be unaware of the group to which the used sealer belonged. Consequently, the inability to conduct a double-masked study is regarded as a significant limitation of this research. Additionally, patients declined to adhere to the control visits due to the completion of root canal treatment in a single session, leading to pain assessment being conducted via telephone communication.

## Conclusion

 Our findings indicate that AH Plus, iRoot SP, and GuttaFlow Bioseal have similar effects on postoperative pain. However, future research should further examine the long-term effects of these different materials and their durability in clinical practice. Additionally, the effects of these materials on periapical healing processes and on critical factors, such as microbial leakage, should be evaluated. A more comprehensive analysis of these data will support clinical decision-making and improve treatment outcomes.

## Competing Interests

 The authors declared that they have no conflicts of interest related to this study.

## Ethical Approval

 All the procedures performed in studies involving human participants were in accordance with the ethical standards of the Institutional Review Board and Ethics Committee of The University of Van Yuzuncu Yil (19–29/04/2021) and with the 1964 Helsinki Declaration and its later amendments or comparable ethical standards. All patients signed an informed consent form after the objectives, procedures, benefits, and potential risks of the study had been explained.
